# Opportunities to improve postpartum care for mothers and infants: design of context-specific packages of postpartum interventions in rural districts in four sub-Saharan African countries

**DOI:** 10.1186/s12884-015-0562-8

**Published:** 2015-06-03

**Authors:** Els Duysburgh, Birgit Kerstens, Seni Kouanda, Charles Paulin Kaboré, Danielle Belemsaga Yugbare, Peter Gichangi, Gibson Masache, Beatrice Crahay, Gilda Gondola Sitefane, Nafissa Bique Osman, Severiano Foia, Henrique Barros, Sofia Castro Lopes, Susan Mann, Bejoy Nambiar, Tim Colbourn, Marleen Temmerman

**Affiliations:** International Centre for Reproductive Health, Department of uro-gynaecology, Ghent University, De Pintelaan 185 P3, 9000 Ghent, Belgium; Institut de Recherche en Sciences et de la Santé, P.O. Box 7192, Ouagadougou, 03 Burkina Faso; International Centre of Reproductive Health Kenya, Obote Avenue - Tudor Estate, P.O. Box 91109, 80103 Mombasa, Kenya; Parent and Child Health Initiative Trust, P.O. Box 31686, Lilongwe, 3 Malawi; International Centre of Reproductive Health Mozambique, Av. Maquiguana, Praceta 1607, Prédio 100, 1 Andar, Maputo, Mozambique; Eduardo Mondlane University, Faculdade de Medicina, Av. Salvador Allende 702, Maputo, Mozambique; Department of Hygiene and Epidemiology, Faculdade de Medicina da Universidade do Porto, Alameda Prof. Hernani Monteiro, 4200-319 Porto, Portugal; Institute for Global Health, University College London, 30 Guilford Street, London, WC1N 1EH UK; Department of Reproductive Health and Research, World Health Organisation, Avenue Appia 20, 1211, Geneva, 27 Switzerland

**Keywords:** Postpartum, Maternal health, Infant, Newborn, Maternal mortality, Health system research, Sub-Saharan Africa

## Abstract

**Background:**

Postpartum maternal and infant mortality is high in sub-Saharan Africa and improving postpartum care as a strategy to enhance maternal and infant health has been neglected. We describe the design and selection of suitable, context-specific interventions that have the potential to improve postpartum care.

**Methods:**

The study is implemented in rural districts in Burkina Faso, Kenya, Malawi and Mozambique.

We used the four steps ‘systems thinking’ approach to design and select interventions: 1) we conducted a stakeholder analysis to identify and convene stakeholders; 2) we organised stakeholders causal analysis workshops in which the local postpartum situation and challenges and possible interventions were discussed; 3) based on comprehensive needs assessment findings, inputs from the stakeholders and existing knowledge regarding good postpartum care, a list of potential interventions was designed, and; 4) the stakeholders selected and agreed upon final context-specific intervention packages to be implemented to improve postpartum care.

**Results:**

Needs assessment findings showed that in all study countries maternal, newborn and child health is a national priority but specific policies for postpartum care are weak and there is very little evidence of effective postpartum care implementation. In the study districts few women received postpartum care during the first week after childbirth (25 % in Burkina Faso, 33 % in Kenya, 41 % in Malawi, 40 % in Mozambique). Based on these findings the interventions selected by stakeholders mainly focused on increasing the availability and provision of postpartum services and improving the quality of postpartum care through strengthening postpartum services and care at facility and community level. This includes the introduction of postpartum home visits, strengthening postpartum outreach services, integration of postpartum services for the mother in child immunisation clinics, distribution of postpartum care guidelines among health workers and upgrading postpartum care knowledge and skills through training.

**Conclusion:**

There are extensive gaps in availability and provision of postpartum care for mothers and infants. Acknowledging these gaps and involving relevant stakeholders are important to design and select sustainable, context-specific packages of interventions to improve postpartum care.

## Background

Postpartum maternal and infant mortality is high in sub-Saharan Africa [1, 2]. Maternal mortality is highest during the first six weeks after birth but remains high throughout the first year after birth due to issues such as untreated anaemia or repeated pregnancies [3, 4]. In 2013, in sub-Saharan Africa, 17.6 % of all maternal deaths occurred intrapartum and the first 24 h postpartum, 47.8 % occurred 24 h to 42 days postpartum, and 13.1 % 43 days to 1 year postpartum [1]. The same year, infant mortality (0–364 days) accounted for 66.9 % of under-5 mortality (deaths between birth and exactly five years of age) in sub-Saharan Africa, and neonatal mortality (0–28 days) for 34.2 % of under-5 mortality [5]. Sub-Saharan Africa accounted for an estimated 49 % (143,380) of all maternal and 51 % (3.2 million) of all under-5 deaths in 2013 [1, 2], while only 12 % of the total female world population [6] and 22 % of the global under-5 population [7] live in sub-Saharan Africa.

The pattern of postpartum mortality and morbidity is clear, but improvement of postpartum care (PPC) as a strategy to enhance maternal and infant health has been neglected [3, 8, 9]. This is also recognised by the World Health Organisation (WHO) which recently published updated guidelines on PPC for mothers and newborns in resource-limited settings in low- and middle-income countries [9]. The guidelines include recommendations on timing, number and place of postpartum contacts, and on contents of PPC for mothers and babies for the first six weeks after birth [9]. However, an essential package of services to support women throughout the first year after childbirth remains poorly defined, and the optimum service delivery configuration and number of routine visits for these services remain unclear [3, 9–12].

Community involvement in improving maternal and infant health in general [13–16] and postpartum care specifically [17, 18] is important. Particularly the establishment of women’s groups trained for and engaged in participatory learning and action taking methods, and the provision of home visits by community health workers, have shown positive effects on maternal and newborn health [13–16, 18]. The importance of upgrading PPC provided at health facilities is stressed in several studies and reports [19, 20]. A combined package including several interventions, adapted to local needs, has the potential to be most effective in improving maternal and child health outcomes [20]. Integration of mother PPC services in child health clinics, which have generally high coverage, is an opportunity to provide the needed but at present often absent PPC for the mother [3]. Involvement of stakeholders and good knowledge of the health system are recognised to be important when designing and introducing new interventions or strategies to improve care [21, 22].

The ‘Missed Opportunities in Maternal and Infant Health’ (MOMI) project focuses on the need to upgrade postpartum care. The project aims to improve maternal, newborn and infant health through improving postpartum care and services by designing, implementing and assessing interventions for the first year after birth in selected rural health districts in Burkina Faso, Kenya, Malawi and Mozambique [23]. The primary objective is to assess the gaps in implementation of evidence-based postpartum care as well as the local context at each site and, on the basis of this, determine how postpartum services can best be organised and provided within existing health systems to improve maternal and infant health outcomes. In our study we define the postpartum period as the period that starts immediately after the birth of the baby and extends up to one year after birth, and PPC as the care provided for mother and infant during this period.

This paper describes the process of designing and selecting suitable, context-specific interventions having the potential to improve PPC.

To design and select the interventions we used the ‘systems thinking’ approach as described by *de Savigny and Adam* [21]. Systems thinking provides a way forward for operating more successfully and effectively in complex, real-world settings. It can open powerful pathways to identifying and resolving health system challenges, and as such is a crucial ingredient for any health system strengthening effort [21]. Regarding intervention design the approach has four steps, being; (1) identify and convene stakeholders, (2) collectively deliberate with stakeholders on proposed interventions and their possible system-wide effects, (3) describe how the proposed interventions will affect health and the health system, and (4) adapt and redesign interventions to optimise positive effects [21].

## Methods

### Study setting

The study took place in four rural districts: Kaya in Burkina Faso, Kwale in Kenya, Ntchisi in Malawi, and Chiúta in Mozambique. Selection of districts was based on being typical for the country in terms of medical infrastructure, equipment and staffing. All levels of care, from community to referral level, were included in the study. Selected demographic and health system related data of the four districts are presented in Table 1.

**Table 1:** District demographic and health system related data

	Kaya district (Burkina Faso)	Kwale district (Kenya)	Ntchisi district (Malawi)	Chiúta district (Mozambique)
**District population**	507,018	162,092	265,470	85,808
*% of women of childbearing age*	22.0	23.5	22.6	21.6
**Number of health facilities**	51	20	12	4
*Primary health care facility*	50	19	11	4
*Referral district hospital*	1^1^	1	1	0
**Median catchment area population per primary health care facility (range)**	9,781 (2,382-25,934)	5,651 (2,944-22,117)	17,141 (7,346-47,794)	21,881 (6,007-36,039)

Source data: Data from the respective district registers: Kenya, January 2012; Burkina Faso and Mozambique, January-December 2011; Malawi, July 2010-June 2011

^1^regional hospital

### Research methodology

Following the ‘systems thinking’ approach we carried out the following four steps of intervention design.

For ‘Step 1’ we conducted a stakeholder mapping to identify the main stakeholders within PPC and their importance in improving PPC. Stakeholders included implementers, managers, policy makers and members of the community and civil society.

For ‘Step 2’ a stakeholders causal analysis workshop was organised at each study site in which the local PPC situation and challenges and possible interventions were discussed [24].

In ‘Step 3’, for each study site, a list of potential interventions was developed by the study researchers. These lists were based on the synthesis of the findings from a comprehensive needs assessment conducted at each research site (Table 2), the inputs from the stakeholders received through the causal analysis workshop and on internationally recognised evidence and guidelines on effective PPC [8, 18, 20, 25–30]. Using all this information local and international MOMI project researchers developed for each study site a list of proposed interventions, described for each of these interventions health system challenges, opportunities and preconditions, and assessed the interventions for their suitability against the following criteria: acceptability, evidence-based, feasibility (taking into account financial and human resources and the availability of infrastructure, medical equipment and drugs), effectiveness, sustainability, and the degree to which the suggested intervention is already included in local maternal, newborn and child health (MNCH) policies (Table 3). A report of this information was compiled for each site [31–34] including a list of provisional recommended interventions to be used to guide stakeholders to agree upon final context-specific intervention packages and adapt to context as appropriate so that positive effects would be maximised and potentially negative effects would be avoided or minimized. This agreement on and selection of final context-specific intervention packages is ‘Step 4’ of ‘systems thinking’ intervention design.

**Table 2:** Needs assessment

The needs assessment included:
1. A critical review of the MNCH policies in the four study countries through [30]:
• document analysis of national, regional and local policies and guidelines,
• semi-structured in-depth interviews with stakeholders at national, regional, and district levels and with health workers, and
• focus group discussions with health workers and women and men from the local community
2. A detailed quantitative situation analysis of existing MNCH services and care at the four study sites using routinely collected and available data on MNCH at national and study site level [29].
Checklist and interview guides to collect needs assessment data were developed. Data collection took place between December 2011 and April 2012 and was performed by project research staff. In-depth interviews and focus group discussions were audio recorded, transcribed and translated if needed. Qualitative data was analysed by extracting themes and triangulating. Quantitative data was entered in an Access database, and checked for errors by the lead partner (SL and HB). A descriptive analysis of the quantitative data was undertaken using SPSS 20.
Results of both baseline studies enabled a description of the national policy landscape in PPC within each setting and evaluation of implementation at local level [29, 30].

MNCH, maternal, newborn and child health

**Table 3:** Development of list of proposed interventions

For each study site the development of a list of proposed interventions included the following process:											
• First, baseline assessment and stakeholders causal analysis workshop PPC findings were summarised in a SWOT analysis. The following characteristics and categories were assessed:											
o Characteristics of postpartum policies – category: postpartum policies											
o Characteristics of postpartum system – categories: health system organization, integration of PPC in other services (child clinic, HIV, FP, etc.), human resources, financial resources, PPC payment modalities for users/clients, and health information system											
o Characteristics of postpartum services – categories: facility-based PP services, community-based PP services, socio-cultural issues and access to PPC, geographic issues and access to PPC, financial issues and access to PPC, and access ‘in time’ to PPC											
o Characteristics of postpartum care – categories: technical effectiveness, patient centeredness, integration, continuity											
*Example Kwale district, Kenya (Characteristics of postpartum services – facility-based PP services)*											
	***Strengths***	***Weaknesses***					***Opportunities***			***Threats***	
***Characteristics of postpartum services***											
**Facility-based PP services**	• none	• PPC is neglected compared to antenatal and childbirth care					• Framework to upgrade facility-based PPC services is available			• Understaffing	
		• Health workers are not aware of importance of PPC								• Lack of interest among health facility staff	
		• Health workers do not know guidelines on PPC									
		• BEmOC services are in part not available at first line health facilities									
• Using the SWOT analysis results and internationally recognised evidence, problems and possible interventions to tackle these problems were identified. Problems were listed for four categories: health system, health services, health care and others.											
*Example Kwale district, Kenya (Care)*											
**Problem identified regarding postpartum care in Kwale district**				**Intervention proposed**							
***Care***											
Attitude of health workers: lack of patient centred care, no respect for cultural beliefs and practices			• Train HWs on patient centred care and culturally appropriate behaviour and approaches								
Quality of care, poor skills of health workers			• Train HWs and establish regular supportive supervision of the HWs by district health management team.								
			• Involvement of district QIT to improve quality of care and HW skills regarding PPC								
Postpartum care not felt as a priority among the health workers			• Sensitize HWs on the importance of PPC and train them on the contents of PPC								
Women discharged less than 24 h after delivery			• Upgrade logistical arrangements in the health HF to enable women to stay at least 24 h after they delivered.								
			• Sensitize HWs and clients on the importance of staying at least 24 h at the HF before being discharged								
• Next the identified possible interventions were described in more detail by mentioning for each the challenges, opportunities and preconditions. Interventions were classified in four groups: (1) community-based interventions, (2) improvement of available PPC services, (3) integration of PPC for the mother in child clinics, and (4) interventions linking the community and health facility.											
*Example Kwale district, Kenya (some interventions on improvement of available PPC services)*											
**Possible Intervention**	**Challenges**				**Opportunities**			**Preconditions**			
***Improvement of available PPC services***											
Improve BEmOC, PPC and other skills of health workers	• Availability of regular supportive supervision				• Availability of QIT to support improvement of quality of PPC			• none			
Train health workers on patient-centred care and culturally adapted behaviour and approaches	• none				• none			• Trainers available			
Sensitisation of health workers on importance of PPC for mother and newborn and PPC training	• Availability of regular supervision to support HWs to deliver PPC				• none			• Make arrangements to enable mother and newborn to stay at least 24 h after delivery			
Dissemination of national guidelines and strategies regarding PPC among the health workers and training on PPC	• none				• Guidelines already available			• none			
• Finally each of the above described interventions was assessed against a set of criteria.											
*Example Kwale district, Kenya (some interventions on improvement of available PPC services)*											
**Possible interventions**	**Criteria** ^**1**^										
	Inclusion in local MNCH policy	Acceptability	Evidence-base	Feasible/ realistic to implement:						Effectiveness	Sustainability (long-term)
				Financial	Human resources	Infrastructure, equipment & supplies	Health system	Referral structure	Supervision		
***Improvement of available PPC services***											
Improve BEmOC, PPC and other skills of health workers	+++	+++	++	+	+	++	++	++	±	++	+
Train health workers on patient-centred care and culturally adapted behaviour and approaches	+	+	+	+	+	+	+		+	+	++
Sensitizing of health workers on importance of PPC for mother and newborn and PPC training	+++	-		++	++	++	+++		+	±	±
Dissemination of national guidelines and strategies regarding PPC among the health workers and training on PPC	+++	++		+	++	++	+++		+++	+	++

^1^The codes range from ‘- - -’ to ‘+++’ to assess the feasibility/relevance of the mentioned criteria

BEmOC, basic emergency obstetric care; FP, family planning; HF, health facility; HIV, human immunodeficiency virus; HW, health worker; PP, postpartum; PPC, postpartum care; QIT, quality improvement team; SWOT, strengths, weaknesses, opportunities and threats

At each study site, Step 4 was conducted through two stakeholders meetings. In the first meeting, the needs assessment results and proposed interventions described in the country reports were discussed. These meetings took place in October 2012 (in Kenya and Mozambique), November 2012 (in Burkina Faso) and February 2013 (in Malawi). A second round of meetings took place in May 2013 in Burkina Faso and Kenya, in July 2013 in Mozambique and in September 2013 in Malawi. At these meetings the final packages of PPC interventions that should be implemented at each study site were agreed upon. Stakeholders decided on the final intervention packages in cooperation with the local study researchers. Stakeholders were free to adapt and select the interventions they wanted, however they had to take care that it was realistic and feasible to implement the selected interventions in the frame of the MOMI study. No financial resources are provided by the MOMI project to maintain and manage the selected interventions.

### Ethics

Written informed consent was obtained from all stakeholders, healthcare providers and community members participating in the in-depth interviews and focus group discussions. To guarantee confidentiality, study tools did not include person identifiers. Ethics clearance was granted by: (1) the Comité d’Ethique pour la Recherche en Santé of the Ministry of Health, Ouagadougou for Burkina Faso; (2) Kenyatta National Hospital, University of Nairobi – Ethics & Research Committee, Nairobi for Kenya; (3) the National Health Science Research Committee, Lilongwe for Malawi; (4) the Comité Nacional de Bioética para a Saude, Maputo for Mozambique; (5) the Ethics Committee ‘Hospital se São João, E.P.E’ Faculdade de Medicina Universidade do Porto for Portugal; (6) the UCL Research Ethics Committee, London, UK; and (7) the Ethics Committee of the University of Ghent, Ghent, Belgium.

## Results

In this section the needs assessment results are summarised first, followed by the results of the intervention design process in each country.

### Needs assessment results

A detailed description of the needs assessment results is given in two project reports [29, 30]. We summarise the findings to show how these supported the design and selection of context-specific PPC interventions.

Results showed that while maternal, infant, and child health is a national priority in all four study countries, specific PPC policy, particularly for maternal health, is weak. All countries use a problem-driven approach to PPC; neither preventive care nor strategies that improve early identification of complications are prioritised. In the four countries, there is very little evidence that interventions known to be effective are implemented [30]. Dissemination of guidance at provincial and district levels is poor in all sites. Awareness of PPC guidelines and policies by healthcare workers is poor and while a minority of women (25 % in Kaya district - Burkina Faso, 33 % in Kwale district - Kenya, 41 % in Ntchisi district - Malawi, 40 % in Chiúta district - Mozambique) receive PPC during the first week after childbirth, at least 70 % of infants attended the health facility for BCG and 85 % for measles vaccination. Involvement of the community and community health workers in PPC is poor. At health facility level, although most facilities provide immediate PPC, women were often discharged before 24 h and provision of the WHO recommended [8, 9] postpartum visits at 72 h and seven days are the exception. Care provision at six weeks was more commonly offered but rarely afterwards, and care in the postpartum period is poorly integrated with other services, such as child immunisation services, family planning clinics and HIV clinics [29, 30].

Little attention was given to the opportunities presented in the postpartum period for effective family planning (FP) at any of the sites, though FP utilisation was highlighted as a priority in all study countries [29, 30]. All facilities except one in Kaya, Burkina Faso, reported offering FP services. In most of these health facilities several FP methods were available, with pills, injectables and male condoms being the most commonly available methods [29]. Despite the availability of these services, demographic and health survey (DHS) data show that the uptake of FP among women of child-bearing age in the respective study districts was low. Following contraception use is reported by the respective DHS: in Burkina Faso’s Central Northern region, in which Kaya district is located, among women aged 15–49 and currently married, 9.5 % use any method of contraception and 9.3 % use a modern method of contraception; in Kenya’s Coast province, in which Kwale district is located, these figures are 34.3 % and 29.7 % respectively; in Malawi 46.1 % and 42.2 %; and in Tete province, Mozambique, in which Chiúta district is located, 15.3 % and 15.1 % [35–38].

Poor organisation of services and quality of care, lack of knowledge in the community, and traditional beliefs and practices further delay or inhibit PPC. Stakeholders and health workers reported understaffing, high staff turnover, poor motivation and lack of staff knowledge and skills on PPC during the causal analysis workshops and the semi-structured interviews. These factors were identified as hampering the provision of good quality PPC [29, 30].

### Selected and agreed upon packages of interventions

At all four sites the intervention packages chosen include interventions to upgrade the PPC provided at the health facilities and the introduction or upgrading of community-based PPC (Table 4).

**Table 4:** Selected interventions in each study district

Study site	Selected interventions
*Burkina Faso – Kaya district*	1. Female community health worker support mother and infant during the postpartum period by:
	• conducting home visits
	• providing individual counselling and group health education on danger signs (for mother and infant)
	• identification of danger signs and referral if needed
	• providing counselling on FP
	2. Upgrade the delivery of immediate postpartum care in the health facilities with focus on the prevention, detection and management of postpartum haemorrhage and sepsis (in mother and newborn) and immediate postpartum FP
	3. Integration of PPC (including FP counselling and provision) for the mother and newborn/infant in the child vaccination clinic
*Kenya – Kwale district*	1. Strengthening immediate postpartum care for mother and newborn by upgrading knowledge and skills of facility and community health workers on detection and management of common maternal and neonatal complications (danger signs counselling, detection and management), promotion of early breastfeeding, counselling and provision of family planning, and by providing postpartum home visits (conducted by the community health worker)
	2. Increase knowledge on and uptake of postpartum family planning during the first year after childbirth using the dialogue model approach at community and facility level.
*Malawi - Ntchisi district*	1. Strengthen clinical management of postpartum care during the postpartum period in the district hospital and health centres (using clinical mentorship and quality of care reviews) with focus on anaemia, sepsis, HIV screening and management, FP and nutrition for the mother and sepsis, pneumonia, feeding and growth monitoring for the infant
	2. Increase utilization of postpartum family planning through awareness raising by providing FP counselling at health facility and community level and by involving males
	3. Strengthen community postpartum care management through home visits conducted by community volunteers and through the establishment and use of men’s, women’s and youth groups. Community volunteers will promote facility-based delivery and provide counselling on nutrition, hygiene, danger signs and FP for the mother and nutrition, immunisation, hygiene and danger signs for the infant.
*Mozambique - Chiúta district*	1. Mother and newborn/infant postpartum risk assessment and management at community and facility level upgraded during the postpartum period through early detection, treatment and referral of postpartum complication cases in health facilities and communities by using a risk assessment checklist. The assessment will focus on following risks, complications and conditions: for mother: sepsis, postpartum haemorrhage, mental/emotional status, anaemia, FP, exclusive breastfeeding and HIV/STI counselling and testing or follow-up, and for infant: sepsis, immunization and growth monitoring, exclusive breastfeeding and HIV/STI exposure.
	2. Scale-up access to and use of family planning through making immediate postpartum IUD insertion available at all district health facilities
	3. Improve access to and use of maternal PPC and services by integrating maternal PPC in child clinics (growth monitoring and immunisation clinics) and by organising quarterly maternal and child health community outreach activities

FP, family planning; HIV, human immunodeficiency virus; IUD, intrauterine device; PPC, postpartum care; STI, sexually transmitted infection

### Kaya district, Burkina Faso

In Kaya district three interventions were chosen: (1) upgrading immediate PPC provided at the health facilities with a focus on detection and management of postpartum haemorrhage and sepsis and immediate postpartum FP, (2) supporting mother and infant during the PP period by female community health workers (CHWs) and (3) integration of PPC in the child vaccination clinics. In order to implement the interventions, all facility health workers involved in providing maternal and child care and all female CHWs will be provided with training backed-up by the provision of guidelines and checklists (written for facility health workers and a picture book for CHWs) on PP care and services. The picture book also serves to provide health education at PPC clients and in the community. To deal with the high staff turnover, yearly refresher training will be organised. To support the implementation quarterly supervision visits of facility and community health workers will be organized in cooperation with the district health team. Information meetings with community leaders and male CHWs will take place to inform them on the project and discuss with them cultural issues and beliefs regarding PPC and FP.

### Kwale district, Kenya

In Kwale district the final selected package of interventions included two interventions: (1) strengthening immediate postpartum care for mother and newborn by upgrading knowledge and skills of facility and community health workers and (2) increasing knowledge on and uptake of postpartum FP during the first year after childbirth. As in Burkina Faso training, distribution of PPC guidelines and regular supervision were chosen as methods of delivering the interventions. To increase postpartum FP uptake, health education sessions using the community dialogue model will be established [39]. Pictures to be used during these sessions will be developed and distributed.

### Ntchisi district, Malawi

In Ntchisi district three interventions were included in final intervention package: (1) strengthening clinical management of postpartum care during the postpartum period in the health facilities, (2) increasing utilization of postpartum FP, and (3) strengthening community PPC management. Unlike the other research sites, in Malawi they chose a system of on-the-job mentorship and training to upgrade PPC knowledge and skills (including knowledge on postpartum FP) of the facility health workers combined with, similar to the other sites, distribution of PPC guidelines and regular supervision visits. To further enhance postpartum FP utilisation, health education sessions with a special focus on involving males will be organised at health facility and community level. Three villages will be selected for implementation of the PPC community intervention. In these villages volunteers will be identified and community women, men and youth groups established. Volunteers will be trained to perform PPC home visits and facilitate community women, men and youth groups. In these groups PPC problems and local feasible solutions for these problems will be identified and discussed.

### Chiúta district, Mozambique

In Chiúta district the final selected package of interventions included three interventions: (1) upgrading mother, newborn and infant postpartum risk assessment and management at community and facility level, (2) increasing access to and use of FP through making immediate PP intrauterine device (IUD) insertion available, and (3) improving access to and use of maternal PPC and services by integrating PPC in child clinics and by organising outreach activities. Training, supervision and the use of specially designed checklists to be completed by the facility or community health worker during each PPC consultation will be used to upgrade PP risk assessment and management. A training session, including skills training, will be organised on PP IUD insertion and availability of IUDs and equipment needed for the insertion will be provided by the authorities. During training and supervision sessions, integration of services will be discussed, monitored and adjusted if needed.

CHWs will not receive a financial incentive at any of the study sites through the project, although non-financial incentives (among others bicycles, T-shirts and gowns, bags) will be used to motivate CHWs and support their work.

## Discussion

The selected interventions are quite similar for the different sites as needs assessment findings were also quite similar for these sites. Overall the interventions proposed focused mainly on increasing the availability and provision of postpartum services and improving the quality of PPC provided through strengthening postpartum services and care at facility as well as community level.

Despite the availability of PPC services in almost all health facilities, similar to other studies in developing countries, including sub-Saharan African countries, in our study sites less than half of the women received PPC within seven days after childbirth [40–42]. Availability of services thus does not guarantee that care is offered. Health workers should also know what kind of care they have to provide, when and why, be skilled and receive the necessary support to enable them to provide this care [43–45]. Like the provision of antenatal care, postpartum care should be offered as a preventive service and not only as a curative service as was found in our study to be often the case.

Our findings of differences between PPC and vaccination coverage are also reported in other studies [41, 46]. The high immunisation coverage may be explained by the fact that immunization schedules are globally fairly well established and therefore conducted more consistently across the sites. Another explanation could be the high level of engagement of different cadres of health care workers in immunization programmes compared to their engagement in PPC services. As found in other studies [47, 48] FP use is low at our study sites, mainly due to persistent social-cultural barriers and limited access to good quality contraception services.

The importance of stakeholders engagement in design and selection of interventions is also highlighted in other studies [49–51]. Similar to these studies we found the investment in long-term and extensive stakeholders involvement very appropriate for the design and selection of interventions as it enhanced ownership, which is crucial for successful and sustainable integration of the intervention in the system [24, 49, 52, 53]. However, the approach considerably slowed the process of finalising the design of the interventions and planning for their implementation. The limited availability of stakeholders and the relatively high turn-over of stakeholders were the main reasons for slowing down the process. We will review the extent to which a sense of ownership of the interventions is maintained by stakeholders throughout the implementation of the interventions.

Compared to the list of provisionally recommended interventions the final packages of interventions selected by the stakeholders included fewer interventions but interventions the stakeholders identified as very needed (based on gaps identified in the needs assessment) and deemed more realistic to implement. Their choice for the final intervention package was largely determined by the fact that interventions had to be implementable within the local available health system and supplies and available financial and human resources. For example, the use of community groups to upgrade PPC when community groups were not already established in the district and setting up outreach PPC services from scratch with implications for financial and human resources to run them, were not selected. The most popular interventions selected were those to improve PPC by upgrading the knowledge and skills of existing cadres (being facility or community health workers) through training and supportive tools such as printed guidelines (text and pictures) or risk assessment checklists and by establishing regular supportive supervision. This selection procedure reasserts the need to take account of local stakeholder knowledge in planning and designing optimum strategies so that service improvements are seen to be practicable and achievable within the local health system context.

As stated above, all the study sites selected interventions to upgrade PPC by involving community health workers. Working with community health workers who do not receive additional financial incentives through the project is challenging. However, as stated by *Bhattacharyya et al.,* contrary to financial incentives, non-financial incentives are critical to the success of any community health workers program [54].

The interventions included in the final context-specific packages are in line with the recommendations on provision and contents of PPC given in the most recently published WHO guidelines [9] and with the national PPC policies of the countries in which the study sites are located [30]. Although in these guidelines and policies, provision of care and the definition of the postpartum period is limited to six weeks after childbirth, other studies address the importance of and opportunities for extending postpartum care for mother and infant beyond six weeks after birth especially for further monitoring of the mother regarding nutrition, anaemia and mental conditions and for enhancing postpartum FP utilisation [3, 4, 55, 56]. The high attendance rates at facilities for newborn and child vaccination highlighted in the needs assessment suggested that integrating maternal and child health services could lead to an increase in PPC and FP provision [57–59]. However, as stated in these studies, to have successful integration of services several preconditions seem to be required including regular technical support, adequate basic working conditions including enough and skilled staff and no drug stock-outs. Service integration could be encouraged through international and national policy drives.

As in other studies [55, 56] the need to increase postpartum FP utilisation to prevent unintended and closely spaced pregnancies was recognised in all study sites as very important but poorly addressed and therefore interventions to increase postpartum FP uptake were selected at all sites.

The process used in our study to design and select context-specific packages of interventions is complex and time consuming but this comprehensive approach is important to enhance the chances of successful intervention implementation [49]. However, additional evaluation of the impact of the selected and implemented interventions on the health of mothers and infants is needed and will be conducted at each study site at the end of the project (from June to September 2015). The effectiveness of the interventions will be evaluated and variables determining effectiveness will be studied. Sustainability and replicability of the selected interventions will also be assessed. This end-of-project evaluation will include qualitative and quantitative research methods.

Although the process described in this paper is complex and time consuming we have shown that it leads to the selection of well-designed, evidence-based and realistic packages of interventions addressing locally identified gaps in PPC.

### Study limitations

The study has some limitations. First, the study is conducted in only one rural district in each country; the PPC situation might differ from other rural districts and from urban areas. Second, because routinely collected and available data on MNCH was used for the needs assessment, not all aspects of PPC could be assessed and at some of the research sites the routinely collected data was of poor quality. Third, limited availability and high turnover of stakeholders meant that the same stakeholders did not always participate throughout the whole intervention design and selection process.

## Conclusion

Strengthening PPC in sub-Saharan Africa is essential to reduce the still unacceptably high postpartum maternal and infant mortality and morbidity in this region. Extensive gaps remain in the availability and provision of PPC for mothers and infants. Acknowledging these gaps and strongly involving relevant stakeholders are important to design and select sustainable, context-specific packages of interventions to improve PPC.
